# Upper Airway Obstruction Elicited Energy Imbalance Leads to Growth Retardation that Persists after the Obstruction Removal

**DOI:** 10.1038/s41598-020-60226-9

**Published:** 2020-02-21

**Authors:** Mohammad H. Assadi, Yael Segev, Ariel Tarasiuk

**Affiliations:** 1 Sleep-Wake Disorders Unit, Soroka University Medical Center, P.O. Box 151, Beer-Sheva, 84105 Israel; 2 Department of Physiology, Faculty of Health Sciences, Ben-Gurion University of the Negev, P.O. Box 105, Beer-Sheva, 84105 Israel; 3 Shraga Segal Department of Microbiology and Immunology, Ben-Gurion University of the Negev, P.O. Box 105, Beer-Sheva, 84105 Israel

**Keywords:** Comorbidities, Respiratory signs and symptoms

## Abstract

Upper airway obstruction can lead to growth retardation by unclear mechanisms. We explored the effect of upper airway obstruction in juvenile rats on whole-body energy balance, growth plate metabolism, and growth. We show that after seven weeks, obstructed animals’ ventilation during room air breathing increased, and animals grew less due to abnormal growth plate metabolism. Increased caloric intake in upper airway-obstructed animals did not meet increased energy expenditure associated with increased work of breathing. Decreased whole-body energy balance induced hindrance of bone elongation following obstruction removal, and array pathways regulating growth plate development and marrow adiposity. This is the first study to show that rapidly growing animals cannot consume enough calories to maintain their energy homeostasis, leading to an impediment in growth in the effort to save energy.

## Introduction

Growth retardation has been frequently reported in children with obstructive sleep apnea (OSA)^1–8^ and conditions of chronic increased upper airway obstruction (AO). It is estimated that 27%–56% of children with OSA will present with growth retardation^1,4,6–8^. To ensure healthy growth, the bones require a significant portion of available fuel^9^. Postulated causes for growth retardation in pediatric OSA include decreased appetite^6,10^, abnormal growth hormone (GH) homeostasis^3^, and increased work of breathing^4^. Several studies found a higher resting metabolic rate in individuals with OSA^11^. Increased upper airway resistance in OSA may promote resting energy expenditure by increasing sympathetic activity and/or the increased work of breathing^12,13^.

Increased AO in rats mimics many of the features of human OSA such as sleep fragmentation and growth impairment^14–17^. Sleep plays an important role in normal growth; AO in children and animals leads to sleep disorder that is associated with reduced GH and global and local epiphyseal growth plate (EGP) insulin-like growth factor 1 (IGF 1), resulting in growth retardation^3,14,17–22^. Orexins play a role in regulation of feeding, sleep, ventilation^23–26^, and sympathetic activity^27,28^. In the AO animals, enhanced hypothalamic orexin, while important in respiratory homeostasis^29,30^, is also responsible for partial sleep loss and sleep disorder^14,15^. Chronically inadequate sleep in adult rats can adversely affect bone metabolism^31^. Plasma concentrations of osteoclast marker tartrate-resistant acid phosphatase 5b (TRAP 5b) increases in sleep restricted rats, leading to bone mass loss. Moreover, orexin neurons can inhibit growth hormone releasing hormone (GHRH) in the hypothalamus and affect sleep^32^. Abnormalities in GHRH underlie both growth retardation and sleep disorder in AO animals^14^. Orexin may also act peripherally through orexin receptor 1 (OX1R) on bone metabolism/architecture by affecting local ghrelin levels^33^. Endochondral ossification is a highly regulated process. Several signaling pathways of ghrelin, including the phosphoinositide 3-kinases/AKT and adenosine monophosphate-activated protein kinase (AMPK) have a key role in chondrogenesis and bone development^34–37^. IGF 1 and its binding protein-2 (IGFBP-2) activate AMPK and bone differentiation^38^. Orexins can stimulate lipogenesis via elevation of peroxisome proliferator-activated receptor gamma (PPARγ) and the PI3K/AKT pathway in marrow adipose tissue^35,39^. The proliferating chondrocytes are regulated Sry-related transcription factor nine (Sox9) that has an important role in chondrogenesis differentiation^39^.

The mechanisms linking whole-body energy balance (i.e., caloric intake vs. energy expenditure) with EGP metabolism/architecture impairment that is AO-induced are poorly understood. We hypothesize that the increased work of breathing leads to abnormal whole-body energy balance and poor development of EGP and linear growth retardation. We used an integrative approach to explore in rapidly growing rats the effects of upper airway obstruction and its removal on whole-body energy balance, EGP metabolism, and linear growth.

In this study, we find that narrowing of the trachea diameter leads to increased energy expenditure and caloric intake, and was not sufficient to meet the energetic demand of breathing. Decreased energy balance leads to the impediment of metabolic processes involved in linear growth that persists after removal of the obstruction. Here, we show that rapidly growing animals cannot consume enough calories to maintain energy homeostasis. Deregulation of energy availability and lack of availability of circulating factors leads to an impediment in linear growth in order to save energy.

## Results

**Figure 1:**
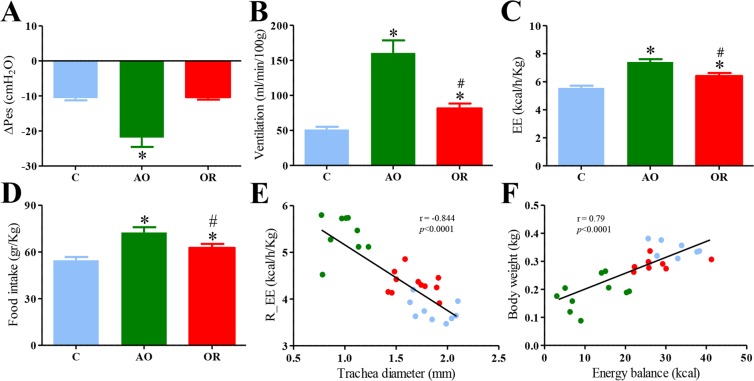
Respiratory and energy metabolism: (**A**) Inspiratory swings in esophageal pressure; (**B**) Minute ventilation; (**C**) Energy expenditure; (**D**) Food intake. Values in B–F were adjusted to effective body mass by ANCOVA analysis; (**E**) Linear regression between R_EE and trachea diameter for each animal; (**F**) Linear regression between body weight and energy balance for each animal. Blue–control, Green–obstructive, Red–obstruction removal, Pes –inspiratory swings in esophageal pressure, EE–energy expenditure. R_EE–Resting EE calculated as mean value for 30-min period with lowest EE. Values are mean ± SEM. *p < 0.01, C vs. AO or OR group. ^#^*p* < 0.01, AO vs. OR. One-way analysis of variance.

Behavior in both the AO and obstruction removal (OR) groups was similar to that of controls. All rat groups were socially active and explored their cages. The trachea diameter was 1.81 ± 0.11 (mm), 0.89 ± 0.05 (mm) (*p* < 0.001), and 1.78 ± 0.04 (mm) (mean ± SD) (*p* = 0.6) for the control, AO, and OR groups, respectively (Supplementary Fig. S1). Over the seven-week observation period, mean (SD) body weight gain was 305 ± 27 (gr), 139 ± 37 (gr) (*p* < 0.001), and 253 ± 30 (gr) (*p* < 0.01) in the control (n = 16), AO (n = 19), and OR (n = 15) groups, respectively. Following tracheal-narrowing, inspiratory swings in esophageal pressure (∆Pes) increased by 206% (*p* < 0.01, Fig. 1A) in the AO group and returned to the control value in the OR group, indicating that resistive loading was produced. Energy expenditure increased by 34.5% in the AO group (*p* < 0.01, Fig. 1C) and remained 16.3% higher in the OR group despite normalization of ∆Pes (Fig. 1A) and trachea diameter (Supplementary Fig. S1**)**. Increased energy expenditure was associated with 37.8% and 14.3% elevation in O_2_ consumption (*p* < 0.001) and increased CO_2_ production by 35% and 13.5% in the AO and OR groups, respectively. The respiratory quotient was similar in all groups (data not shown). Increased CO_2_ production was associated with 313% (*p* < 0.01) and 58% (*p* < 0.01) elevation of ventilation during room air breathing in the AO and OR groups, respectively (Fig. 1B**)**. Food intake was 32.6% (*p* < 0.001) and 13.7% (*p* < 0.01) higher in the AO and OR groups, respectively (Fig. 1D). Energy balance was 29.1 ± 1.97 (kcal), 11.6 ± 2.03 (kcal) (*p* < 0.01), and 27.1 ± 1.81 (kcal) in the control, AO, and OR groups, respectively. Trachea diameter inverse correlated with resting energy expenditure (r = −0.84, *p* < 0.0001, Fig. 1E), and energy balance correlated with body weight (r = 0.79, Fig. 1F).

**Figure 2:**
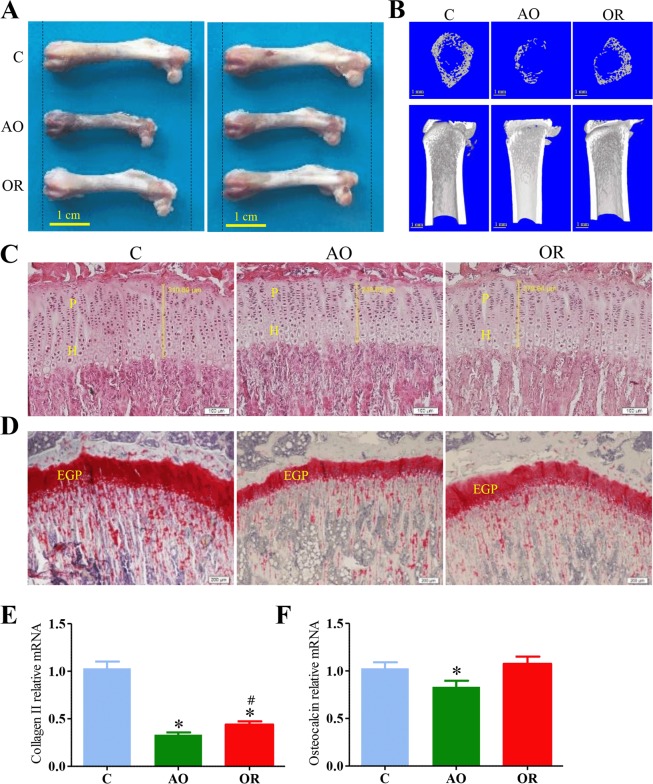
Somatic growth and bone architecture. (**A**) Photograph of femur in two animals in each group; (**B**) Three-dimensional reconstructed Micro CT image of the trabecular and the entire proximal femur; (**C**) Growth plate stained with hematoxylin and eosin. Vertical yellow bar indicates growth plate width; (**D**) Growth plate stained with Safranin O stain; (**E**) Collagen type II mRNA level; (**F**) Osteocalcin mRNA level; C–control (blue), AO–upper airway obstruction (green), OR–obstruction removal (red), EGP–epiphyseal growth plate, H–Hypertrophic, P–Proliferative. *p < 0.05, C vs. AO or OR group. ^#^*p* < 0.05, AO vs. OR. One-way analysis of variance.

Decreased EGP width in AO and its partial improvement following OR (Table 1, Fig. 2C) were associated with reduced bone elongation (Fig. 2A). Tibia length was 38.5 ± 0.55 (mm, n = 14), 31 ± 0.48 (mm) (*p* < 0.01, n = 19), and 36.3 ± 0.32 (mm) (*p* < 0.01, n = 15) for the control, AO, and OR groups, respectively. A three-dimensional reconstructed micro-computed tomography (CT) image of a distal trabecular femur indicated that the AO group decreased bone development and OR partially improved growth (Fig. 2B, Table 1). The trabecular bone volume to total volume (BV/TV) ratio decreased by 34.8% and 15.6% (*p* < 0.01) in the AO and OR groups, respectively. Trabeculae number (Tb.N) decreased by 26.8% and 11.5% (*p* < 0.01) in the AO and OR groups, respectively, while trabecular separation (Tb.Sp) increased by 38.9% (*p* < 0.01) in both groups. Cortical BV/TV ratio decreased by 10% (*p* = 0.01) in both the AO and OR groups. Cortical bone mineral density (BMD) decreased by 8.6% and 7.3% (*p* = 0.01) in the AO and OR group, respectively. Safranin O showed decreased staining intensity of the primary spongiosa in the AO group and was only partially improved following OR (Fig. 2D). Serum TRAP 5b was undetected in all groups. Collagen II mRNA expression was reduced by 67% and 57% in the AO and OR groups, respectively (*p* < 0.05; Fig. 2E). Osteocalcin mRNA expression was reduced by 17% in the AO group (*p* < 0.05, Fig. 2F).

**Table 1:** Bone architecture and micro-computed tomography analysis at 7 weeks.

	Control	AO	OR
Tb.BV (mm^3^)	9.74 ± 0.85	4.57 ± 0.51*	6.73 ± 0.4*^,#^
Trabecular BV/TV (%)	37.38 ± 2.14	24.37 ± 2.53*	31.55 ± 1.6*^,#^
Tb.N (1/mm)	2.35 ± 0.08	1.72 ± 0.14*	2.08 ± 0.07*^,#^
Tb.Sp (mm)	0.36 ± 0.02	0.5 ± 0.02*	0.48 ± 0.04*
Cortical BV (mm^3^)	2.96 ± 0.06	1.9 ± 0.14*	2.5 ± 0.06*^,#^
Cortical BV/TV (%)	52.23 ± 1.18	46.96 ± 1.8*	48.64 ± 0.84*
Cortical BMD (g/cm^3^)	0.69 ± 0.01	0.63 ± 0.01*	0.64 ± 0.01*
Total EGP width (μm)	306 ± 7.6	246 ± 6.8*	274 ± 4.5*^,#^
Proliferative EGP width (μm)	172 ± 4.2	135 ± 4.7*	152 ± 2.6*^,#^
Hypertrophic EGP width (μm)	134 ± 5.0	111 ± 2.6*	121 ± 2.8*^,#^

EGP–epiphyseal growth plate; BMD–Cortical bone mineral density, BV–bone volume, Cortical BV/TV–bone volume/tissue volume, Tb.Bv–Trabecular number/Bone volume, Tb.N–Trabecular number, Tb.Sp–Trabecular separation, Trabecular BV/TV–bone volume/tissue volume, TV–Tissue volume. Control (n = 11); AO–obstructive (n = 9); OR–obstruction removal (n = 10).

*p < 0.01, C vs. AO or OR.

^#^*p* < 0.05, AO vs. OR.

One-way analysis of variance.

**Figure 3:**
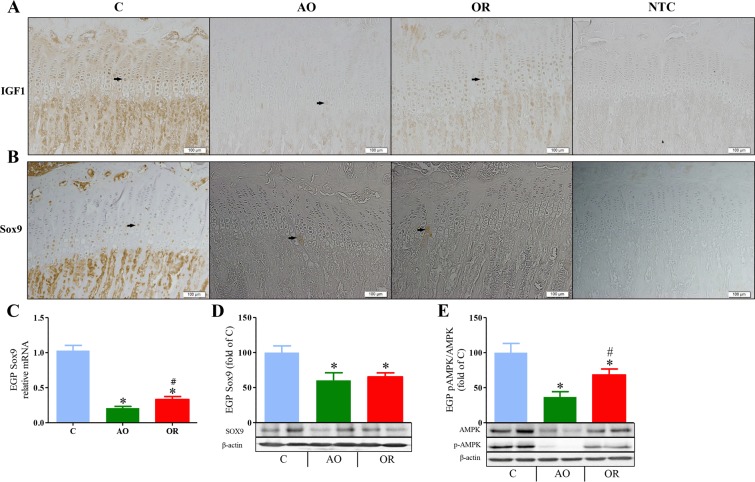
Growth plate development markers. (**A**) IGF-1 protein distribution in the epiphyseal growth plate tested by immunohistochemistry (n = 8 in the control and AO groups and n = 6 in the OR group); (**B**) Sox9 protein distribution in the epiphyseal growth plate tested by immunohistochemistry (n = 8 in each group). (**C**) Epiphyseal growth plate Sox9 mRNA level (n = 7 in each group); (**D**) Growth plate Sox9 protein determination by western blot analysis (n = 8 in each group); (**E**) Epiphyseal growth plate pAMPK/AMPK protein determination by western blot analysis (n = 8 in each group). Arrows in A and B point to positive stained cells, Bar = 100 μM. C–control (blue), AO–upper airway obstruction (green), OR–obstruction removal (red), IGF1– Insulin-like growth factor 1, Sox9–Sry-related transcription factor nine EGP–epiphyseal growth plate, AMPK–AMP-activated protein kinase; pAMPK–phosphorylation of AMPK. *p < 0.01, C vs. AO or OR group. ^#^*p* < 0.01, AO vs. OR. One-way analysis of variance.

**Figure 4:**
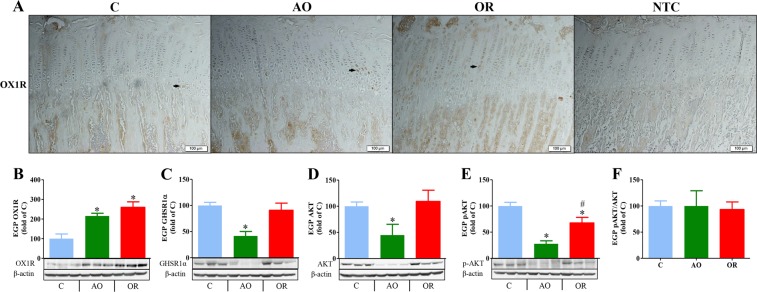
Growth plate OX1R, GHSR1a, and AKT expression. (**A**) OX1R protein distribution in the epiphyseal growth plate tested by immunohistochemistry, Bar = 100 μM (n = 8 in each group). Arrows point to OX1R positive stained chondrocytes; (**B**) Epiphyseal growth plate OX1R protein determination by western blot analysis (n = 8 in each group); (**C**) Epiphyseal growth plate GHSR1a protein determination by western blot analysis (n = 6 in each group); (**D**) Epiphyseal growth plate AKT protein determination by western blot analysis (n = 6 in each group); (**E**) Epiphyseal growth plate phosphorylation of AKT (p-AKT) determination by western blot analysis (n = 6 in each group); (**F**) p-AKT/AKT ratio (n = 6 in each group). OX1R–Orexin receptor 1, EGP–Growth plate, GHSR1a–growth hormone secretagogue receptor 1a. *p < 0.01, C vs. AO or OR group. ^#^*p* < 0.01, AO vs. OR. One-way analysis of variance.

The mean number and range of EGP IGF 1 positive cells, as determined by immuno-histochemistry (Fig. 3A and Supplementary Fig. S2A), were 56 (46–65 range), 3 (1–6 range), and 54 (37–74 range) in the control, AO, and OR groups, respectively. IGF-binding protein 2 (IGFBP2) mRNA was 1.075 ± 0.101, 0.41 ± 0.033 (*p* < 0.001), and 0.69 ± 0.058 (*p* < 0.001) fold of control in the control, AO, and OR groups, respectively. The EGP transcription factor Sox9 immunohistochemistry (Fig. 3B and Supplementary Fig. S2B), mRNA (Fig. 3C), and protein (Fig. 3D) were significantly reduced in both the AO and OR groups. The mean number and range of Sox9 positive cells were 18 (5–30 range), 1 (0–2 range), and 2.5 (1–4 range) in the control, AO, and OR groups, respectively. The mean EGP pAMPK/AMPK ratio decreased by 63% (*p* < 0.001) and 31.8% (*p* < 0.01) in the control, AO, and OR groups relative to the control group, respectively (Fig. 3E). OX1R protein in the EGP was higher in the AO group and did not normalize in the OR group (Fig. 4A,B and Supplementary Fig. S2C). The mean number and range of OX1R positive cells were 15 (11–25 range), 30 (27–33 range), and 33 (27–44 range) in the control, AO, and OR groups, respectively. Growth hormone secretagogue receptor 1a (GHSR1a) protein was reduced 58.4% in the AO group (*p* < 0.01, Fig. 3D). EGP AKT protein (Fig. 4D) and phosphorylation of AKT (P-AKT) (Fig. 4E) were significantly reduced by 56% and 73%, respectively, in the AO group (*p* < 0.01). No change in the P-AKT/AKT ratio was observed in either the AO or OR group (Fig. 4F).

**Figure 5:**
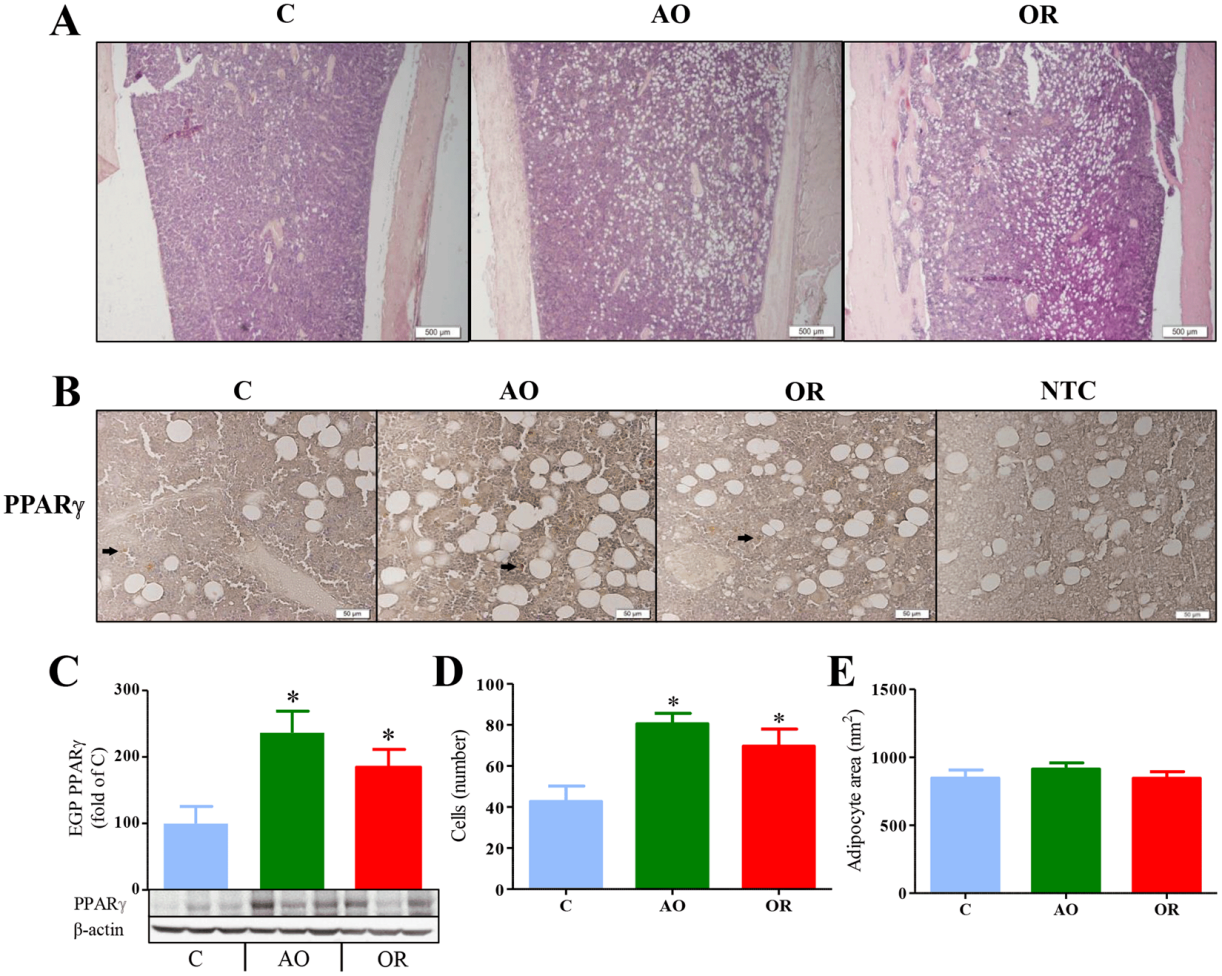
Marrow adipocytes. (**A**) Marrow adipocyte cells – Movat’s modified pentachrome staining. Bar = 500 μM (n = 7 in each group); (**B**) PPARγ protein distribution in the growth plate tested by immunohistochemistry. Arrows point to positive stained cells. Bar = 500 μM; (**C**) PPARγ protein determination by western blot analysis; (**D**) Marrow adipocyte cell number in square mm (n = 8 in each group); (**E**) adipocyte area (n = 8 in each group). PPARγ–peroxisome proliferator-activated receptor gamma. Bar = 500 μM (n = 8 in each group). *p < 0.01, C vs. AO or OR group. One-way analysis of variance.

Both AO and OR groups had increased marrow adipose cell numbers (Fig. 5A,D). Marrow PPARγ protein increased by 236% (*p* < 0.01) and 184% (*p* < 0.01) in the AO and OR groups, respectively (Fig. 5B,C**)**. The mean number and range of PPARγ positive cells were 3 (2–4 range), 17 (6–28 range), and 10 (7–13 range) in the control, AO, and OR groups, respectively (Fig. 5B). No significant change was found in the marrow adipose cell cross-sectional area (Fig. 5E).

## Discussion

The effects of AO and its removal on energy balance and EGP development/metabolism were explored from weaning to adulthood. Increased caloric intake in the AO group was not sufficient to meet the energy demand of the increased work of breathing; thus, energy balance (i.e., caloric intake vs. energy expenditure) was reduced close to 60%. This study is the first to show that decreased energy balance led to an impediment in bone elongation and bone development. The EGP growth impediment was associated with an alteration in the array of metabolic processes involved in development, including IGF 1, Sox9, AMPK, and OX1R. Despite the normalization of energy balance in OR, only partial improvement of EGP growth and metabolism were observed. Bone requires a substantial portion of the available fuel and nutrients to generate ATP for proper devlopment and growth^40^. Increased adiposity in the bone marrow was associated with upregulation of PPARγ and loss of bone mass. Our findings indicate that AO leads to abnormal function of the stroma for hematopoietic cell differentiation. Here, we show that rapidly growing animals cannot consume enough calories to maintain energy homeostasis; this deregulation of energy availability and lack of availability of circulating factors leads to impediment in EGP growth/architecture in order to save energy.

### Upper airway obstruction and energy expenditure

Untreated OSA may lead to growth retardation^1–4,7,8,18,41–43^, and bone mass loss in adults^5^ by mechanisms that are poorly understood. To the best of our knowledge, this study is the first to show that deregulation of energy homeostasis plays an important role in AO-induced growth retardation. Increased energy expenditure in AO is due to upregulation of ventilation in order to maintain respiratory homeostasis^30,44,45^ and the extra energy needed for increased additional wakefulness^30^. Orexin participates importantly in maintaining respiratory homeostasis in AO, but it is also responsible for partial sleep loss^29^. Decreased EGP width and bone growth in AO were associated with reduced mRNA expression of type II collagen, the main component of cartilage^46^ and osteocalcin^47^, respectively. Interestingly, serum osteoclast marker TRAP 5b was undetected in our study. Insufficient sleep over a long duration can lead to poor bone health that is associated with increased TRAP 5b and decreased osteoblast activity^31^. AO animals increased their caloric intake due to elevation of gut-derived ghrelin, an array of hypothalamic mediator factors, and orexin^30^. Orexin and ghrelin-containing neurons could influence each other, and thereby regulate feeding behavior^48^, while short sleep *per se* also can stimulate gut-derived ghrelin and feeding^49^. Although caloric intake increased, the slow body weight gain strongly indicates the higher energetic cost of airway obstruction, and AO animals could not consume enough calories to meet the additional energy requirements to maintain energy homeostasis – a condition that is not associated with malabsorption of calories^15,50^. Tissue composition of water, protein, and fat in the vital organs of AO are largely spared, and the slow body weight gain was mostly related to reduced adiposity tissue^15^. In contrast, food restriction resulting in weight loss is associated with severe organ weight losses^51^.

Earlier studies in children demonstrated that the z score for weight correlates with sleeping energy expenditure in OSA because of the increased work of breathing during sleep^1^. AO in rats, on the other hand, was both inspiratory and expiratory, and may be representative of upper airway resistance patients’ that is not exclusively sleep related (i.e., patients with increased nasal resistance, subglottic or tracheal stenosis, retrognathia, or macroglossia). In OSA, airway obstruction during sleep is intermittent with opportunity for recovery during the day^2,43^. The amount of oxygen consumed by the respiratory muscles is about 1% to 2% of the basal VO_2_^52^. Increased resistive breathing and a variety of other pulmonary diseases can substantially increase the energetic cost of breathing. Regulation of energy balance depends on factors such as basal metabolic rate, thermic effect of food, and caloric intake^11,53,54^. OSA may also elevate energy expenditure by increasing sympathetic activity^11–13^. An increased firing rate of the sympathetic nerves to brown adipose tissue through β-adrenergic receptors increases energy expenditure to generate heat. However, this possibility is unlikely since AO animals do not respond to administration of norepinephrine to generate heat due to decreased brown adipose uncoupling protein 1, leptin level, and lack of available fuel for heat generation^30^.

### Growth plate metabolism/architecture

AO-induced growth retardation, and despite the normalization of energy balance in OR, only partial improvement in tibial length and EGP width was observed. The skeleton in highly metabolic active tissues requires substantial amounts of energy, particularly during periods of rapid growth and physical activity^40,55,56^. Bone formation requires available fuel and communicates metabolic demands to other metabolically active tissues via circulating factors^40^. Further studies are needed to explore the effects of shorter obstruction duration and longer recovery period on growth velocity to determine if growth retardation is largely irreversible. Growth retardation was associated with a large suppression of an array of metabolic processes that are involved in EGP and hematopoietic cell differentiation. The IGF system plays an important role in early longitudinal growth by acting both as an endocrine and as an autocrine/paracrine close to the site of synthesis^20^. IGF1 with IGFBP2, one of the main EGP and bone IGFBPs, stimulate AMPK activation and osteoblast differentiation, and AMPK knockout mice have reduced bone mass^37^. OR normalized EGP IGF 1 cell number; however, IGFBP 2 mRNA and AMPK were not normalized. Moreover, pharmacologically restoring local EGP IGF 1 level only partially restored growth in AO animals^14,22^. This indicates that other pathways are involved in addition to the GH/IGF1 axis in this growth retardation. In mice, OX1R could regulate ghrelin content locally in the bone^33^. In our study, elevation of OX1R in the EGP was associated with partial improvement in EGP architecture following OR. Several signaling pathways of ghrelin play a role in chondrogenesis and osteoblastogenesis^33,57,58^. Moreover, OX1R is suppressed during osteoblast differentiation and elevated during adipocyte differentiation^33^. In this study, reduction of growth pate GHSR1α was associated with abnormal EGP architecture and growth retardation. Interestingly, EGP OX1R did not improve in the OR group, while GHSR1α was similar to the control. This finding may suggest that in EGP, GHSR1α and OX1R are independently regulated. Further studies are needed to explore the effects of sleep and caloric restriction on EGP OX1R. We found increased adipose cell number and marrow PPARγ, an essential element in adipocyte differentiation processes in many tissues^59^. Orexin activates PPARγ in the marrow and is associated with bone mass loss and increased adipogenesis^33^. Sox9 is important in chondrogenesis differentiation^60^.

### Summary

In this study, we showed that rapidly growing animals cannot consume enough calories to maintain energy homeostasis. This deregulation of energy availability is associated with a considerable impediment in EGP metabolism/architecture and abnormal growth in order to save energy. Thus, surgical intervention *per se* may not be sufficient to prevent growth retardation, and endocrine correction with one or more availability circulating factors may be essential.

## Methods

### Animals

This study was approved by the Ben-Gurion University of the Negev Animal Use and Care Committee protocol number IL-40-07-2018. All protocols comply with the American Physiological Society Guidelines. Male 22-day-old Sprague-Dawley rats (48–55 gr) were used. Animals were kept on a 12–12 light–dark cycle with lights on 09:00 at 23 ± 1.0 °C. Animals were given food (3272 Kcal/kg) and water *ad libitum*.

### Surgery

The technique used for sham surgery and to induce AO in juvenile rats was as previously described^14,15,17,47^. During the surgical procedures, the mortality rates in the AO and OR group were less than 10%, and an additional 5% mortality was observed 2–5 days after surgery. Data were collected on days 45–49 post surgery. Following surgery, prophylactic enrofloxacin 5 mg/ml (s.c.) and water containing ibuprofen (0.1 mg ml^−1^) were given for three days^14,15,17,30^.

### Experimental schedule

In the current study, we used a previously described experimental schedule (Supplementary Fig. S3)^17,30^. On day 14, the AO group was randomized, and OR of the silicon band was performed on half of the animals. Measurements of respiration, energy metabolism, and the effect of propranolol energy metabolism were performed on days 45–48. On day 49, animals were sacrificed, serum was collected and tissue was extracted after death and stored at −80 °C.

### Respiratory and energy metabolism

Respiratory activity at room air was recorded by whole body plethysmography (Buxco, DSI, St. Paul, MN, USA). ∆Pes was measured from a saline-filled catheter placed in the lower third of the esophagus and connected to a pressure transducer^22,30,44,45^. Seven animals were used to analyze esophageal pressure. Metabolic activity was measured using a behavioral phenotyping system (Sable Instruments, Las Vegas, NV, USA), as previously described^30,61^. Animals were allowed a 24 h acclimation period followed by a 48 h sampling duration. Energy expenditure was calculated as VO_2_ × (3.815 + 1.232 × respiratory quotient), and was normalized to effective body mass. Resting energy expenditure was calculated as the mean value for a 30-min period with lowest energy expenditure. The respiratory quotient was calculated as the ratio of CO_2_ produced by O_2_ consumed by the body.

### Histology

Five-μm thick longitudinal sections were cut and collected on Superfrost™ Plus slides for histology staining of Safranin O^15^, hematoxylin, and eosin^22^. Total growth-plate width and proliferative and hypertrophic zone widths were measured as previously described (n = 8 in each group)^15,29^. Trachea histology photomicrographs were obtained by light microscope, with the internal border of the trachea outlined and cross-sectional area and diameter calculated for each animal, as previously described^17,30^.

### Immunohistochemistry

Immunohistochemistry staining was performed using a protocol described previously by our laboratory (n = 6 in each group)^15^. Anti-rabbit OX1R, anti-mouse IGF-1 (Abcam, Cambridge, MA, USA), anti-mouse PPARγ and anti-mouse Sox9 (Santa Cruz Biotechnology, Santa Cruz, CA, USA) were used for immunohistochemistry staining. For image processing, Cellsens Entry software (MATIMOP, Tel Aviv, Israel) was used. All of the experiments and observations were repeated at least three times.

### Quantitative real-time PCR

Assays were performed with power SYBR green PCR master mix (Applied Biosystems) as previously described^15,29^ using the ABI Prism 7300 Sequence Detection System (Applied Biosystems).

### Tissue and serum and biomarker measurements

The following antibodies were used for evaluation of the EGP extracts by western immunoblot^15,17,29^: GHSR1a (n = 6 in each group), Sox9 (n = 8 in each group), PPARγ (n = 6 in each group) (Santa Cruz Biotechnology, Santa Cruz, CA, USA), OX1R (n = 8 in each group) (Abcam, Cambridge, MA, USA), AKT (n = 6 in each group), p-AKT (n = 6 in each group), AMPK (n = 8 in each group), p-AMPK (n = 8 in each group) (Cell Signaling Technology, Danvers, MA, USA), and β-actin (MP Biomedical Solon, OH, USA). The full protocol is described in our previous publication^29^. Serum TRAP 5b was measured using an ELISA kit (MBS704438; MyBioSource, Inc., San Diego, CA, USA). The low and high detection limits were 0.312 and 40 mlU/ml, and the coefficient of variation was <5%.

### Micro CT scanning and analysis

Femurs were scanned using a μ-CT machine (Skyscan 1174 v2. Bukner, Kontich, Belgium) at 27-µm resolution using a 0.25-mm aluminum filter following protocol as previously described by our laboratory^15^.

### Data analysis

One-way analysis of variance was used to determine the significance between groups. The correlations between trachea diameter and resting energy expenditure, energy balance, and body weight were assessed by linear regression. Null hypotheses were rejected at the 5% level.

## Supplementary information

Supplementary Information.
