# Supplementary material for: Upper Airway Obstruction Elicited Energy Imbalance Leads to Growth Retardation that Persists after the Obstruction Removal
Source: Sci Rep. 2020 Feb 21;10:3206. doi: 10.1038/s41598-020-60226-9 (PMC7035324; doi:10.1038/s41598-020-60226-9)
Supplement: Supplementary file 1 — Supplementary Information. [file 41598_2020_60226_MOESM1_ESM.doc]

# Supplementary Information

Upper Airway Obstruction Elicited Energy Imbalance Leads to Growth Retardation that Persists after the Obstruction Removal

Mohammad H. Assadi, Yael Segev, and Ariel Tarasiuk

**Supplementary methods**

**Animals**

This study was approved by the Ben-Gurion University of the Negev Animal Use and Care Committee protocol number IL-40-07-2018. All protocols comply with the American Physiological Society Guidelines. Male 22-day-old Sprague-Dawley rats (48–55 gr) were used. Animals were kept on a 12–12 light-dark cycle with lights on 09:00 at 23±1.0ºC. Animals were given food and water *ad libitum.*

**Surgery**

The technique used for sham surgery and to induce AO in juvenile rats was as previously described.^1-7^ Animals were anesthetized with tribromoethanol (200 mg kg^-1^) administered intraperitoneally (i.p.). A midline ventral cervical incision was made, and the trachea exposed and dissected so as not to damage adjacent structures. A circumferential silicon band 0.5-cm long was placed around the trachea to induce tracheal narrowing (**Supplementary Fig. 1**). Controls underwent sham surgery with no tracheal narrowing. On day 14, the AO group was randomized and obstruction removal (OR) of the silicon band was performed, and the remaining AO animals underwent a second sham surgery (**Supplementary Fig. 2**).^6,7^

**Metabolic activity and food intake**

Indirect calorimetry and food intake were measured using the Promethion High-Definition Behavioral Phenotyping System (Sable Instruments, Inc., Las Vegas, NV, USA).^7^ Data acquisition and instrument control were performed using MetaScreen software V.2.3.14.6, and the obtained raw data were processed with ExpeData version 1.9.14, using an analysis script detailing all aspects of data transformation. Respiratory gases were measured by the GA-3 gas analyzer (Sable Systems, Las Vegas, NV, USA) using a pull-mode, negative-pressure system. Air flow was measured and controlled by FR-8, with a set flow rate of 2500 ml/min. Water vapor was continuously measured, and its dilution effect on O_2_ and CO_2_ was mathematically calculated. Animals had free access to food and water, and were allowed a 24 h acclimation period followed by a 48 h sampling duration. Effective mass was calculated by ANCOVA analysis, as described previously.^8,9^ Respiratory quotient (RQ) was calculated as the ratio between CO_2_ produced to O_2_ consumed. During measurements, body weight and food intake were monitored daily.

### Respiratory activity

Respiratory activity was recorded by whole body plethysmography (Buxco, DSI, St. Paul, MN, USA). Inspiratory swings in esophageal pressure (∆Pes) were measured in anesthetized animals.^1,3,4,7^ Esophageal pressure was measured from a fine saline-filled catheter placed in the lower third of the esophagus and connected to a pressure transducer.

## Micro CT scanning and analysis

Femurs were scanned using a μ-CT machine (Skyscan 1174 v2. Bukner, Kontich, Belgium) at 27-µm resolution, using a 0.25-mm aluminum filter. X-ray tube peak potential was 50 kV, and X-ray intensity was 800 µA. Analysis of trabecular bone was performed along 2 mm, starting 0.4 mm below the distal growth plate's border (n= 9 controls and n= 8 AO). Each image was reconstructed from 498 slices 0.2 μm in width.^5^Structural indices were calculated using Skyscan CT Analyzer (CTAn) software. A set of two hydroxyapatite (HA) phantoms were scanned and used for calibration of bone mineral density (BMD). Trabecular bone volume per total volume (BV/TV), mean trabecular thickness (Tb.Th), mean trabecular number (Tb.N), and mean trabecular separation (Tb.Sp) indices were computed using a marching cubes algorithm. For cortical bone, 2 mm in the direction of the metaphysis were taken as an offset from the GP, and a 1-mm area was chosen for cortical bone analysis. Cortical BV/TV and BMD were calculated.

**Western immunoblot analysis**

Hypothalamus tissue was homogenized on ice with a polytron (Kinetica, Littau, Switzerland) in lysis buffer (50mM Tris, pH 7.4, 0.2% Triton X-100) containing 20mM sodium pyrophosphate, 100mM NaF, 4mM EGTA, 4mM Na3VO4, 2mM PMSF, 0.25% aprotinin, and 0.02 mg/mL leupeptin). Extracts were centrifuged for 20 min at 17,000 g at 4°C, and the supernatants were collected and frozen. Homogenates were mixed with 4× sample buffer and boiled for 5 min. Then 100-μg portions of sample protein were loaded in each gel lane and subjected to 7.5–15% SDS polyacrylamide gel, and electroblotted into nitrocellulose membranes. Blots were blocked for 1 h in TBST (0.05% Twin-20) buffer (10mM Tris, pH 7.4, 138mM NaCl) containing 5% non-fat dehydrated milk, followed by overnight incubation with primary antibodies. The band antibody was visualized by enhanced chemiluminescence (ECL; Biological Industries, Beit Haemek, Israel), and images were taken using a Microchemi 4.2 (DNR Bio Image System, Jerusalem, Israel). Densitometric analyses were performed using Image J.^2,5^

## RNA extraction and real time-PCR

## Total RNA was extracted from the hypothalamus using the PerfectPure RNA Tissue kit (5 PRIME, Hamburg, Germany), and cDNAs were synthesized using a high-capacity cDNA reverse transcription kit (Quanta Biosciences, Beverly, MA, USA). Quantitative real-time PCR (qPCR) assays were performed with power SYBR green PCR master mix (Quanta Biosciences) using the ABI Prism 7300 Sequence Detection System (Applied Biosystems, Foster City, CA, USA). Primers for quantification of Soc9, IGFBP2, Type II collagen, Osteocalcin and β-actin (n =8 in each group) (Sigma-Aldrich, Rehovot, Israel) are summarized in Table S1. Each sample was analyzed in triplicate in individual assays. The specificity of the reaction is given by the detection of the melting temperatures (Tms) of the amplification products immediately after the last reaction cycle. The target gene expression value was calculated by the ΔΔct method after normalization with a housekeeping gene (β-actin).^2,3,6^

**Supplementary References**

1. Tarasiuk, A. Scharf, S. M., & Miller, M. J. Effect of chronic resistive loading on inspiratory muscles in rats. *J. Appl. Physiol.* **70**, 216-222 (1991).
2. Segev, Y., Berdugo-Boura, N., Porati, O. & Tarasiuk, A. Upper airway loading induces growth retardation and change in local chondrocyte IGF-I expression is reversed by stimulation of GH release in juvenile rats. *J. Appl. Physiol*. **105**, 1602–9 (2008).
3. Tarasiuk, A., Berdugo-Boura, N., Troib, A., & Segev, Y. Role of GHRH in sleep and growth impairments induced by upper airway obstruction in rats*. Eur. Respir. J.* **38**, 870-877 (2011).
4. Tarasiuk, A., Levi, A., Berdugo-Boura, N., Yahalom, A., & Segev, Y. Role of orexin in respiratory and sleep homeostasis during upper airway obstruction in rats. *Sleep* **37,** 987-998 (2014).
5. Tarasiuk, A., Levi, A., Assadi, M. H., Troib, A., & Segev, Y. Orexin plays a role in growth impediment induced by obstructive sleep breathing in rats. *Sleep* **39,** 887-897 (2016).
6. Assadi, M.H., Shknevsky, E., Segev, Y. &Tarasiuk, A. Abnormal growth and feeding behavior persist after removal of upper airway obstruction in juvenile rats. *Sci. Rep.* **7**: 2730 (2017).
7. Assadi, M.H., Segev, Y. & Tarasiuk, A. Irreversible metabolic abnormalities following chronic upper airway loading. *Sleep* doi: 10.1093/sleep/zsz176.
8. Mina, A.I. *et al.* Web-based analysis tool for indirect calorimetry experiments. *Cell Metab.* **28,** 656-666 (2018).
9. Tschöp, M.H. *et al.* A guide to analysis of mouse energy metabolism. *Nat. Methods.* **9,** 57-63 (2011).

**Supplementary Figure 1**: **Representative photograph of trachea in three animals in each group.** Arrows in the obstructive group indicate the point where cartilage rings of the trachea “folded in,” generating the site of obstruction. Following removal of the obstruction, the trachea returned to its normal native shape. Surgeries were performed at a young age when trachea cartridge was still “flexible” and able to return to its normal shape.

**Supplementary Figure 2: Image with higher magnification**. (A) Insulin-like growth factor 1 IGF1; (B) Sox9; (C) OX1R; Arrow points to positive stained cell; C–control, AO–upper airway obstruction, OR – obstruction removal. IGF1–Insulin-like growth factor 1; OX1R–orexin receptor 1.

**Supplementary Figure 3: Flow diagram of study groups and times data were collected**.


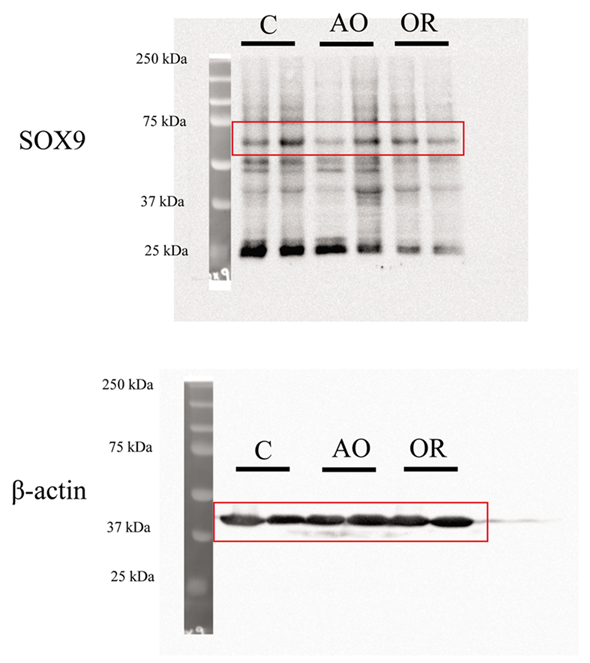


**Supplementary Figure 3**: **Full-length original western blots for SOX9 referred to in Figure 3.**


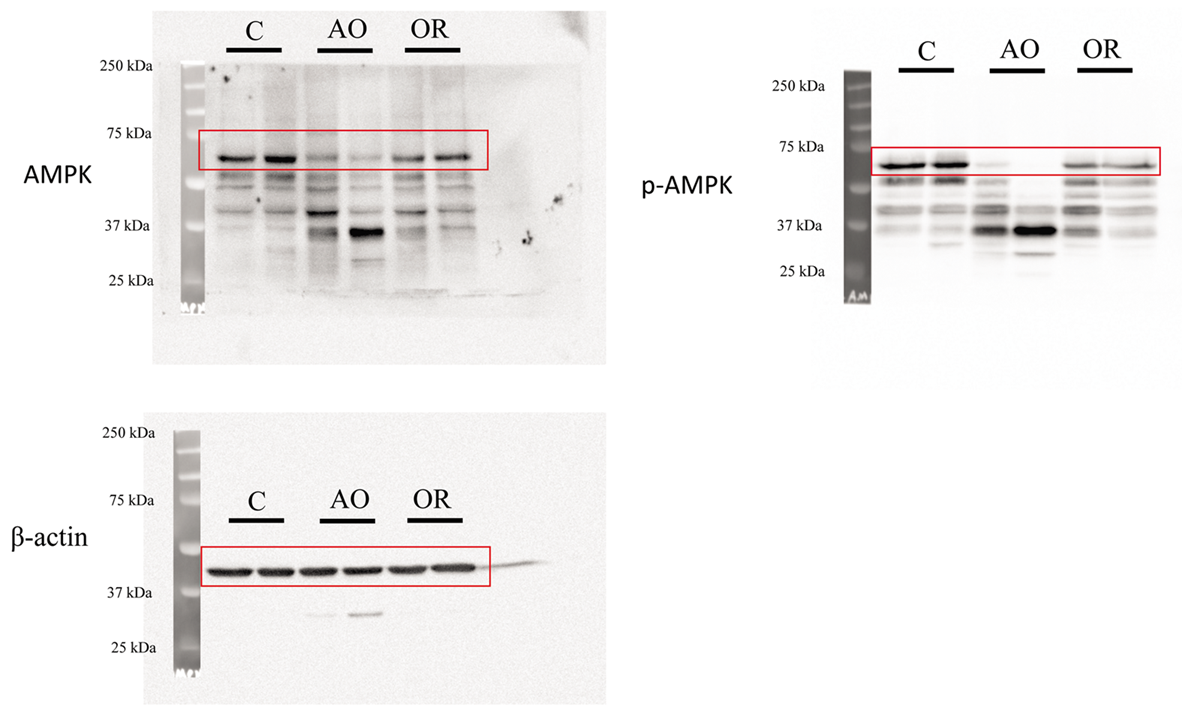


**Supplementary Figure 4**: **Full-length original western blots for AMPK and p-AMPK referred to in Figure 3.**


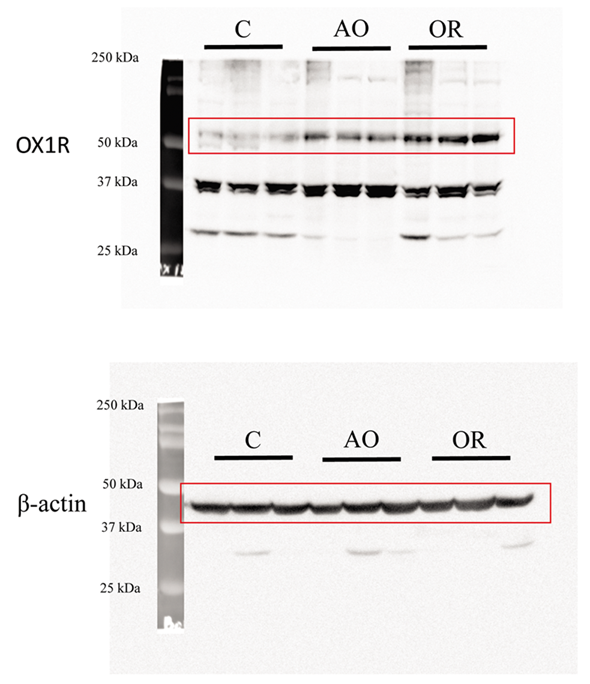


**Supplementary Figure 5:** **Full-length original western blots for OX1R referred to in Figure 4.**


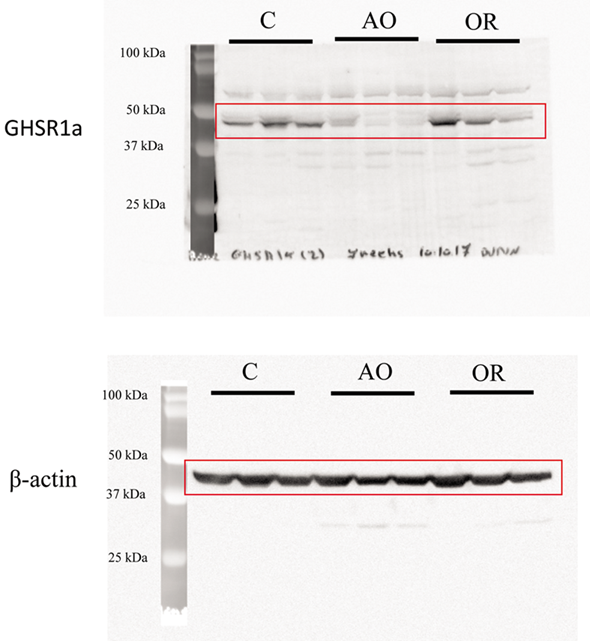


**Supplementary Figure 6:** **Full-length original western blots for GHSR1α referred to in Figure 4.**


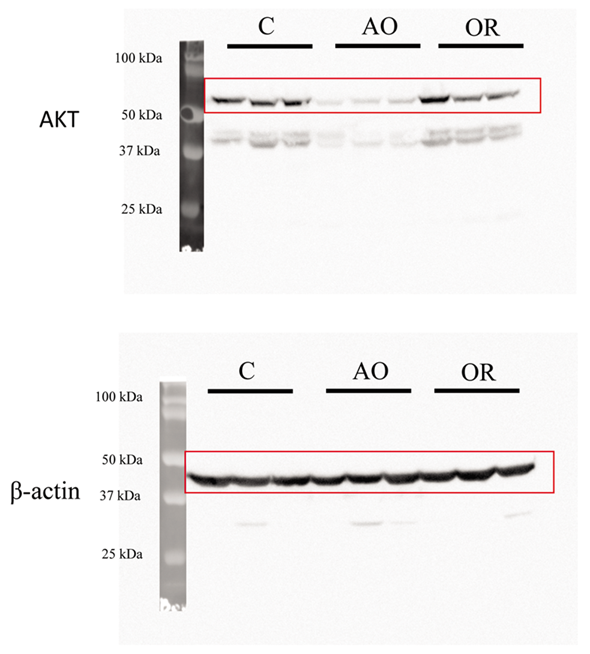


**Supplementary Figure 7**: **Full-length original western blots for AKT referred to in Figure 4.**


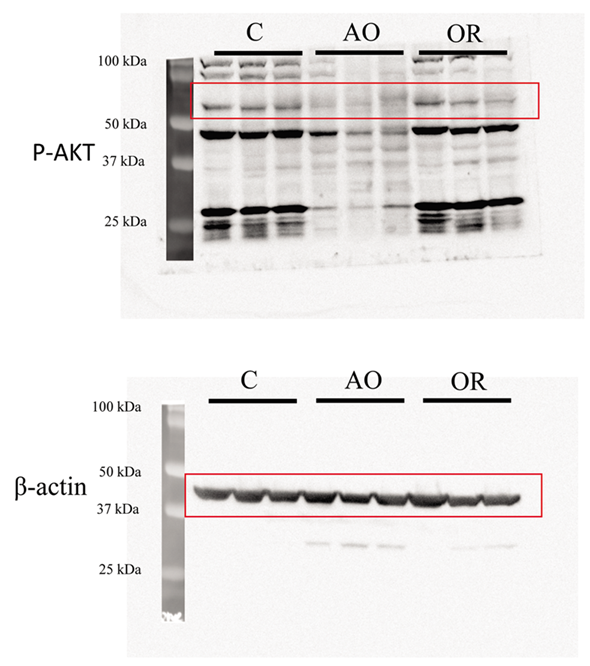


**Supplementary Figure 8**: **Full-length original western blots for p-AKT referred to in Figure 4.**


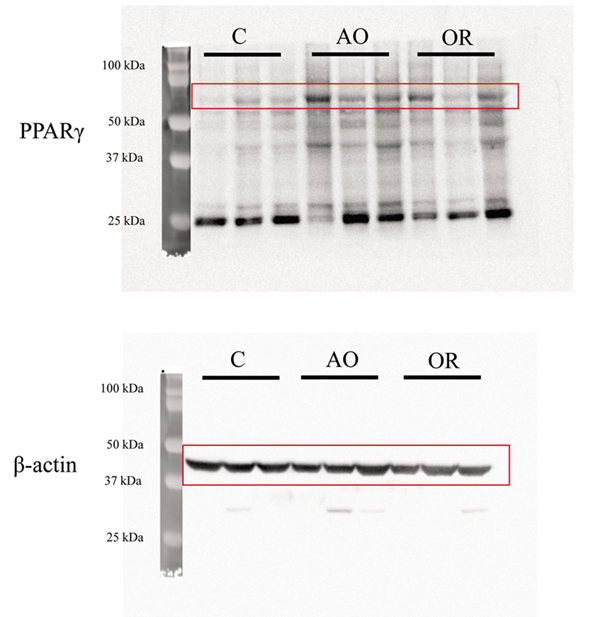


**Supplementary Figure 9**: **Full-length original western blots for PPARγ referred to in Figure 5.**

|  | **Forward primer** | **Reverse primer** |
| --- | --- | --- |
| Sox9 | CGTCAACGGCTCCAGCA | TGCGCCCACACCATGA |
| IGFBP2 | AAGGCGCATGGTGGAGAT | CCCTAAGTCAGGCATGAAGG |
| Type II collagen | CCCAGAACATCACCTACCAC | GGTACTCGATGATGGTCTTG |
| Osteocalcin | GAGCTAGCGGACCACATTGG | CCTAAACGGTGGTGCCATAGA |
| β-Actin | CGTCATCCATGGCGAACT | CCCGCGAGTACAACCTTCT |

**Supplementary Table S1:** Primer sequences used for genes studied

Sox9–Sry-related transcription factor nine; IGFBP2–insulin-like growth factor binding protein 2
